# Solid Right Ventricular Compression by Intraventricular Septum-Hematoma Induced after Percutaneous Coronary Intervention

**DOI:** 10.1155/2016/6218723

**Published:** 2016-03-28

**Authors:** Ibrahim El-Battrawy, Ibrahim Akin, Benedikt Münz, David Manuel Leistner, Michael Behnes, Thomas Henzler, Holger Haubenreisser, Theano Papavassiliu, Martin Borggrefe, Ralf Lehmann

**Affiliations:** ^1^First Department of Medicine, University Medical Centre Mannheim (UMM), Faculty of Medicine Mannheim, University of Heidelberg, Theodor-Kutzer-Ufer 1-3, 68167 Mannheim, Germany; ^2^German Center for Cardiovascular Research (DZHK), Partner Site Mannheim, Mannheim, Germany; ^3^Department of Clinical Radiology and Nuclear Medicine, University Medical Centre Mannheim (UMM), Faculty of Medicine Mannheim, University of Heidelberg, Theodor-Kutzer-Ufer 1-3, 68167 Mannheim, Germany

## Abstract

Intraventricular septum-hematoma is a rare complication following percutaneous coronary intervention (PCI). This complication may represent a challenge for accurate diagnosis and treatment. This case report is about a 60-year-old male patient being admitted with an acute coronary syndrome. Despite successful PCI with drug eluting stent implantation into the right coronary artery (RCA) the patient complained about recurrent angina pectoris according to Canadian Cardiovascular Society (CCS) class IV. Cardiac magnetic resonance imaging and transthoracic echocardiography revealed a massive 4.9 × 9.2 cm sized end-diastolic septum-hematoma, which compromised right ventricular cavity. Emergent recoronary angiography ruled out further contrast extravasation from the RCA. Conservative treatment was intended after discussion in the “heart-team.” The patient completely recovered with nearly complete resolution of the hematoma after 6 months.

## 1. Introduction

Intraventricular septum-hematoma is a rare life-threatening sequela, usually associated with myocardial infarction, chest trauma, and complication of percutaneous coronary intervention [1–3]. It can also occur spontaneously [4, 5]. Intraventricular septum-hematoma may lead to significant morbidity and mortality. No systematic review regarding management of this complication is available.

## 2. Case Report

**Figure 1:**
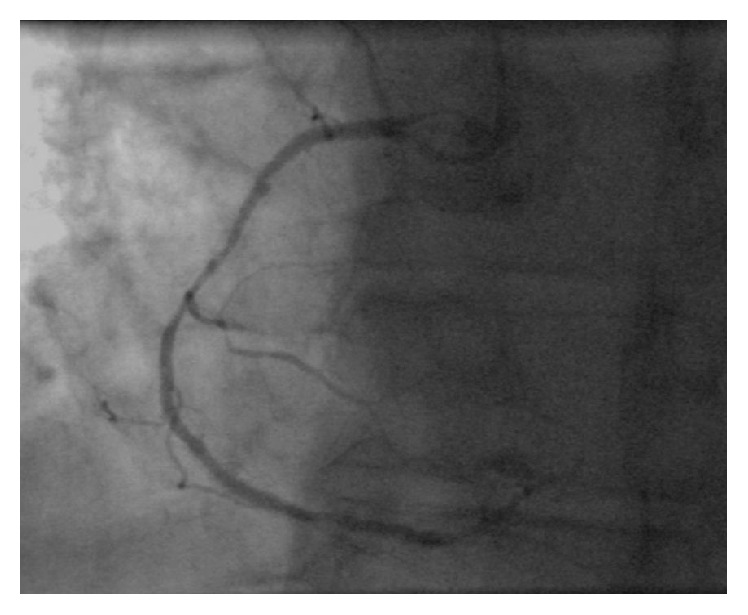
Coronary angiography after admission revealed long 90% diameter stenosis within the prestented right coronary artery (RCA), which was determined as the culprit lesion.

**Figure 2:**
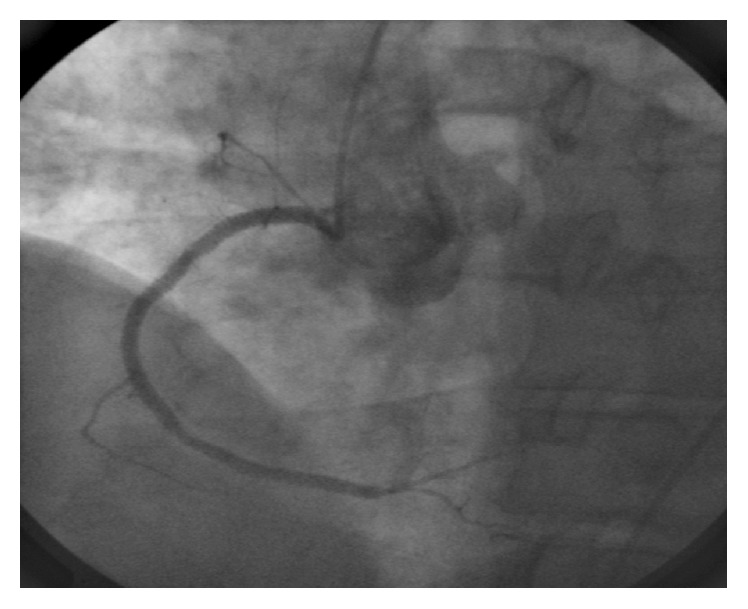
Coronary angiography after predilation and implantation of four drug eluting stents in the right coronary artery showed acceptable result.

We report the case of a 60-year-old male patient, who was hospitalized with an acute coronary syndrome (ACS) in a referral hospital and transferred to our facility for acute percutaneous coronary intervention (PCI). Coronary angiography over the right radial approach revealed a severe 3-vessel coronary artery disease with diffuse moderate stenosis of left anterior-descendens (LAD), ramus circumflexus (LCX), and long 90% diameter stenosis within the prestented right coronary artery (RCA), which was determined as the culprit lesion (Figure 1). PCI was complicated by the long calcified lesion and a restricted backup despite using an Amplatz I (AL1) 6F guiding catheter over the radial access. The initial placed Runthrough guide wire (Terumo®) was subsequently changed to a 300 cm stiff wire (Iron Man, Abbott Vascular®) along with a 1.5/12 mm Mini Trek® over-the-wire balloon (Abbott Vascular) to improve PCI-support. Four drug eluting stents (DES, Promus Element 3.5/24 mm, Promus Element 3.0/38 mm, Promus Element 3.0/16 mm, and Promus Element 2.75/38 mm; Boston Scientific, Natick, Massachusetts, USA) were overlapped implanted after predilatation. The angiographic result was acceptable (Figure 2) and the patient free of complaints. However, final angiogram revealed a little coronary perforation in a small end-branch (diameter < 1 mm) of the ramus interventricularis posterior (RIVP, see Video-File 1 in Supplementary Material available online at http://dx.doi.org/10.1155/2016/6218723) of the RCA with prompt washout of the contrast-medium without intramyocardial staining. Additional echocardiographic exclusion of pericardial effusion was performed. Therefore, the finding was interpreted as an iatrogenic intracavitary fistula, and a conservative management without specific intervention was determined.

**Figure 3:**
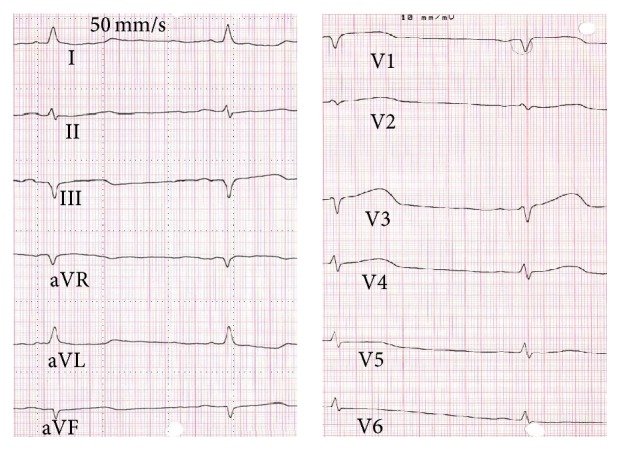
ECG after the index PCI revealed increased transient ST-elevation in the anteroseptal leads V2–V5.

**Figure 4:**
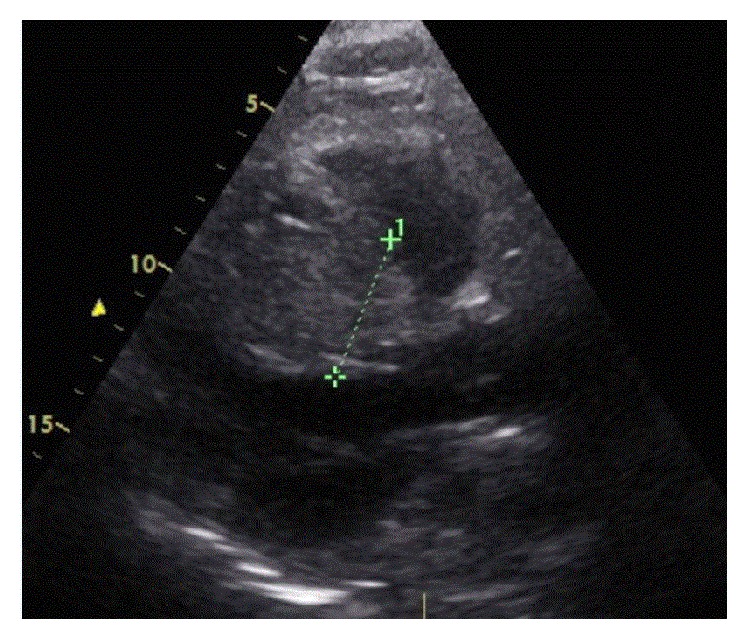
Echocardiogram shows a huge swelling of the intraventricular septal wall with compression of the right ventricle.

**Figure 5:**
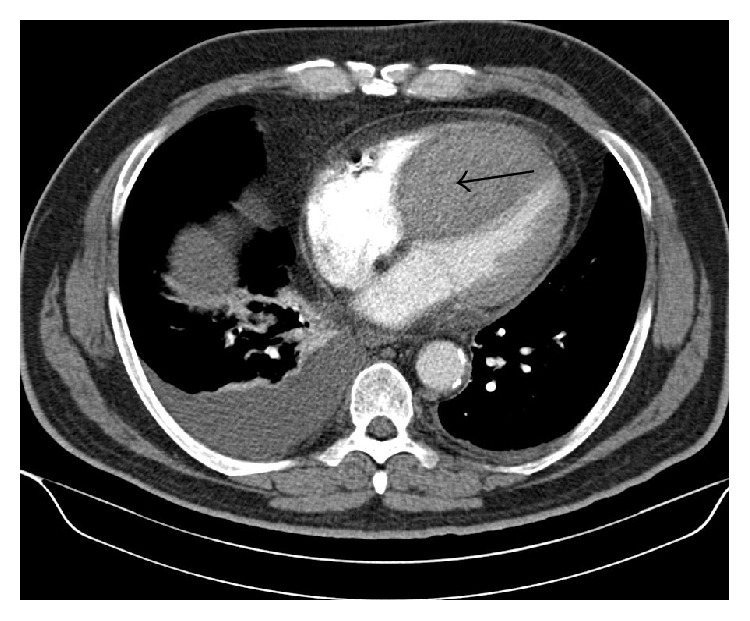
CT scan confirms a huge intraventricular septum-hematoma.

**Figure 6:**
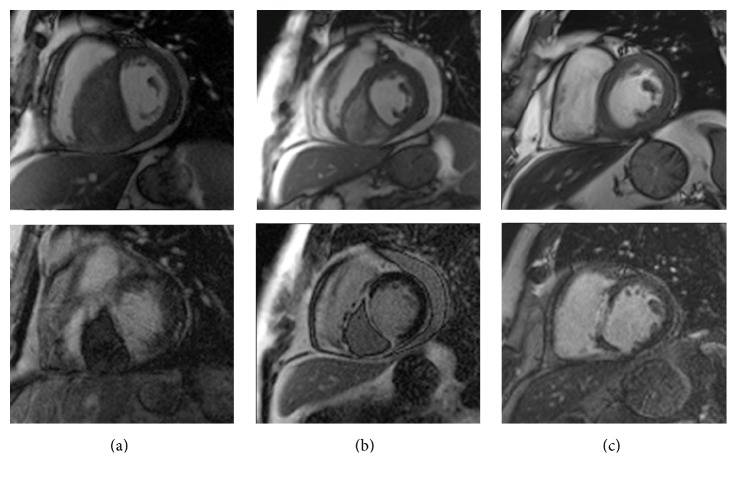
Follow-up CMR after one month (b) and 6 months (c) revealed a nearly complete resolution of the hematoma, top row: end-diastolic phase of a 4D cine-SSFP sequence of a basal slice in a short axis view, bottom row: corresponding late gadolinium enhancement images. LGE imaging revealed a transmural LGE with a central intramural hypointensity, corresponding to an intramural haematoma extending from the septal to the inferior wall, from the base to the apex.

Because of increased transient ST-elevation in the anteroseptal leads V2–V5 (Figure 3) and recurrent angina pectoris second-look angiography was performed one hour after the index procedure, which confirmed the final result of index procedure in the RCA and spasm of the medial LAD with resolution after intracoronary administration of 0.2 *μ*g nitroglycerin with accompanying ST-resolution and chest pain regression. 24 hours later patient complained of chest pain again. Emergency echocardiography (Figure 4) showed a huge swelling of the intraventricular septal wall with compression of the right ventricle and congestion in the hepatic veins, but no pericardial effusion. An intraventricular septum-hematoma was suspected and coronary angiogram ruled out persistent contrast streaming from the RCA potentially through compression of the crux cordis by the septum mass. Initial ACT was 236 seconds at index PCI and 5000E ProAmatine were administered at the second-look angiography. Initial perforation was not recognized during index PCI. Computer tomography (CT) and cardiac magnetic resonance (CMR), for better evaluation of myocardial tissue with regard to vitality, confirmed solid (4.9 × 9.2 cm end-diastolic; Figures 5 and 6(a)) intraventricular septum-hematoma with obliteration of the right ventricular cavity. Left ventricular function was moderately deceased (EF = 42%). Conservative treatment was intended after discussion in the “heart-team” due to the hemodynamic stable situations regardless of subtotal compression of right ventricle. The surgeons were restrictive due to the pathology of the septum being not consistent enough resulting in limited possibility for resection and suture, and the patient was monitored for eight weeks with initial heart failure symptoms NYHA III and slow advance after initiation of a heart failure therapy including diuretics. Due to potential arrhythmogenic effects of the hematoma and decreased ventricular ejection fraction patient was provided with a life vest (LifeVest® Zoll-Lifecor, Pittsburgh, USA).

Fortunately, follow-up echocardiography as well as CMR after one month, Figure 6(b), and two months and six months, Figure 6(c), presented a nearly complete resolution of the hematoma and decreasing obliteration of the right ventricular cavity. Left ventricular ejection fraction improved from 42% to 47%. Nevertheless late enhancement technique revealed a septal myocardial scar. Congestive heart failure state improved from NYHA III to NYHA I, and heart failure therapy and life-vest-protection were determined.

## 3. Discussion

This case report demonstrates a severe unexpected complication of initial assumed small coronary perforation, which was treated conservatively. Due to the large number [6] of inapparent coronary perforations during PCI, it is questionable if such small perforations justify routine balloon blockage of the proximal vessel or injection of prothrombotic agents for example air, thrombin, and adrenaline until perforation is fixed. The main potential risks of such maneuvers were ischemia in further myocardial segments and thrombus formation in the recently implanted coronary stents.

Nevertheless a possible reason of persistent or recurrent angina after successful coronary revascularization could be intramyocardial bleeding. Therefore, a dedicated echocardiography beyond the routine work-up with ECG and cardiac enzymes should be performed routinely in patients with recurrent angina after PCI.

Development of intramyocardial hematoma is a very a rare complication after PCI [3, 4]. No systematic review regarding management of this complication is available. On the other hand, spontaneous remission has been reported in a few cases [4, 5]. This complication may present a diagnostic and treatment challenge. Echocardiography, CT, and CMR are helpful. The possible reason for this complication in the presented case might be the stiff coronary guide wire despite the floppy tip.

In conclusion, we suggest that a conservative approach of intramyocardial hematoma is the therapeutic approach of choice in patients who are clinically and hemodynamically stable. Close follow-up and monitoring in the intensive care unit are necessary.

## Supplementary Material

Final coronay angioram demonstrated a little coronary perforation in a small end-branch of the ramus inventricularis posterior of the RCA with prompt washed-out of the contrast-medium without intramyocardial staining.
